# Obsessive–compulsive existential type: a dialectical-phenomenological approach

**DOI:** 10.3389/fpsyg.2023.1211598

**Published:** 2023-09-05

**Authors:** Lívia Fukuda, Melissa Tamelini, Guilherme Messas

**Affiliations:** ^1^Faculdade de Ciências Médicas da Santa Casa de São Paulo, São Paulo, Brazil; ^2^Instituto de Psiquiatria, Hospital das Clínicas, Faculdade de Medicina, Universidade de São Paulo, São Paulo, Brazil; ^3^The Collaborating Center for Values-Based Practice, St Catherine’s College, Oxford, United Kingdom

**Keywords:** obsessive–compulsive existential type, obsessive–compulsive disorder, dialectical phenomenological psychopathology, anthropological disproportions, phenomenology, dialectics

## Abstract

The clinical presentation of obsessive–compulsive patients is characterized by unwanted, intrusive, nonsensical, self-related, and recurrent ideas, thoughts, images, or impulses associated with active compulsive compensations. Under the operational diagnostic criteria adopted by the biological- and cognitive-oriented neopositivist medical paradigm, it is known as “obsessive–compulsive disorder.” However, this paradigm has been criticized for its controversial assumptions, limited methodologies, theoretic biases, and inconsistency in producing practical outcomes. To bypass some of these issues, we propose a complementary approach that draws on and further develops existing psychopathological studies of the obsessive–compulsive anthropological condition based on dialectical phenomenological psychopathology. As such, we refer to the global clinical configuration as the “obsessive–compulsive existential type.” Our theoretical inspiration comes from the classical phenomenological work on obsessions undertaken by Straus and Gebsattel, which identified the negative transformation of the obsessive–compulsive life-world or the endogenous emergence of the *anti-eidos* (diluting existential force). We then propose to broaden the concept of *anti-eidos*, especially in its dialectical correlation with *eidos* (unifying existential force), representing the existential dialectic between transformation and permanence. Next, we detail the dynamics of anthropological disproportions in obsessive–compulsive existential type, essentially the supremacy of the *anti-eidos* over the *eidos*. This primary imbalance modifies the obsessive–compulsive existential structure, consisting of polymorphic temporality; weakened intentionality; maladjusted calibration of distance with the world and others; an integral, isolated, besieged self with dwindling self-agency, and tense and over-protecting embodiment. We also analyze compensatory hyperreflexivity and compulsive rituals as expressions of structural counterbalancing designed to contain the primary structural disproportions and derangements. The heterogeneous obsessive–compulsive clinical manifestations are the complex result of the primary structural alteration and subsequent phenomenological compensations. They tend to be variable in temporal span and rarely assume a fixed form, hindering diagnosis. We correlate structural frameworks with multiple clinical examples. Finally, we raise some insights on how our study may contribute to scientific research and therapeutic proposals.

## 1. Introduction

There is a typical clinical presentation in which individuals experience their consciousness as being invaded by thoughts, ideas, images, or impulses that they consider their own, but which are incongruent or senseless and occur recurrently with imperative force over long periods (Jaspers, 1913/1997; Binder, 1936/1979; American Psychiatric Association, 2013). This phenomenon is called obsession and is generally related to a state of anxiety, suffering, and tension, as well as sensory phenomena (such as autonomic discharge, disgust, tactile impressions of being dirty, and feelings of incompleteness or “not just right”; Janet, 1903; Bragdon et al., 2021). Some common themes of these obsessions include contamination, concern about causing harm or hurting, aggressiveness, blasphemy, sexual taboos, symmetry, and hoarding (Stein et al., 2019). The individual feels overpowered, out of control, with a depleted sense of freedom (De Haan et al., 2015). To mitigate, offset, or eliminate such obsessions and suffering, individuals may be compelled to perform compensatory behaviors or mental acts called compulsions involving, for example, rituals, magical thinking, or prayers (Jaspers, 1913/1997; American Psychiatric Association, 2013). The most frequent rituals are washing, checking, counting, and organizing (Stein et al., 2019). Obsessive–compulsive individuals can decline into a vicious cycle of obsessions and compulsions, and many go undiagnosed or delay seeking treatment because they feel ashamed of their symptoms (El-Sayegh et al., 2003; Hezel et al., 2022). This condition can lead to distress and impairment, impacting both personal and professional life and burdening public health worldwide (Torres et al., 2017).

Descriptions of this presentation can be traced back to religious and literary accounts from the 17th century and medical accounts from the 19th century (Berrios, 2008). Such descriptions have remained stable to the present day in different regions (Horwath and Weissman, 2000) and cultures (Williams et al., 2017). How this condition has been understood and studied, and the names it has been given have varied according to the ontological and epistemological assumptions of the psychiatric paradigms in vogue. Nowadays, with the predominance of biological and cognitive models and criterion-based diagnosis, the nosological term used to refer to this condition is obsessive–compulsive disorder (OCD).

Despite the extensive research on the genetic, neurobiological, and neuropsychological characteristics of OCD in recent decades, the translation of these findings into clinical practice and patient care has been slow (Kapur et al., 2012; Dumas-Mallet and Gonon, 2020; Troisi, 2022). This limited correspondence between research findings and practical outcomes seen in the current neopositivist medical model can be explained by its lack of accuracy in delineating the object of study, controversial selection of assumptions, theoretic biases, and limited methodologies (Huda, 2019; Wilshire et al., 2021). The ontological and epistemological choices made in any research can significantly impact the validity of its empirical findings (Parnas et al., 2013). When controversial choices are made, the resulting empirical studies may unintentionally reinforce questionable assumptions and lend unwarranted support to reductionist theories, treating them as absolute (Aragona, 2011; Wilshire et al., 2021). If we aim to completely understand obsessive–compulsive conditions, we should look at the qualitative and interconnected aspects of the symptoms, individual differences, and clinical variety. This requires us to go back to the basics of our practice, which is the study of psychopathology, and remove any preconceptions or theoretical biases (Messas and Fulford, 2021; The Lancet Psychiatry, 2021; Ritunnano et al., 2023).

This article delves into the obsessive–compulsive anthropological condition, which we call the obsessive–compulsive existential type. Through the lens of dialectical phenomenological psychopathology (Messas et al., 2022), we aim to analyze its subjective, intersubjective, and objective characteristics comprehensively. In this heuristic method, the experiential field is given precedence, prioritizing knowledge derived directly from patient-psychopathologist interactions, thereby illuminating the structure of consciousness and the dynamics of its formal determinants in each individual structure. We chose this methodology because we believe it is coherent with the complexities and transformations of the nature of the psychiatric object, enabling the study of the essential modification, the progression into the pathological process, and the compensatory mechanisms designed to contain the primary condition. By tracing the changes that lead to the development of the disorder, we can gain insight into its natural progression and provide some initial contributions to therapy and research in this field.

Before analyzing the obsessive–compulsive existential type, we will briefly introduce dialectical phenomenological psychopathology.

## 2. Dialectical phenomenological psychopathology

Phenomenology and dialectics were incorporated into psychopathological studies by Jaspers in his work, *General Psychopathology* (Jaspers, 1913/1997). For Jaspers, phenomenology—static understanding—was a method to describe and classify altered subjective experience without preconceptions or theoretical frameworks. Since 1922, Jaspers’ legacy has been critically enriched by the phenomenological psychopathology’s authors investigating the fundamental constituents of particular experiences (Binswanger, 1923/2006; Minkowski, 1933/1995; Straus, 1935; Gebsattel, 1938/1966). The essential aprioristic determinants of the conditions of possibility of particular experiences are temporality, spatiality, intersubjectivity, identity, and corporeity, which configure an indivisible totality referred to as existential structure^1^ (Tamelini and Messas, 2017; Messas et al., 2018). This unique configuration exists only when instantiated individually, relying on its participative dependence on the surrounding world and others. In this sense, we remain within the scope of anthropology (Kraus, 2001). In every case, the psychopathologist looks for typical alterations of the pre-reflexive determinants constituting specific psychopathological frameworks (Fuchs, 2010). When the structure becomes stable and fixed, it can be recognized through an act of eidetic reduction (Kraus, 2001) or empathic penetration (Minkowski, 1933/1995) as the essential core of the pathology (Messas, 2021). Fixation is often observed when the alteration strikes the deepest foundations of the existential structure (i.e., pre-reflective, tacit level of selfhood, or minimal self). The paradigmatic structural pathology is schizophrenia, defined as disturbed ipseity (Sass, 2010).

As phenomenological psychopathology evolved, it became clear that merely identifying a static, fixed essence is limited because it fails to take account of the historical and open nature of human existence and the dynamism of the structure toward both a distorted and fixed pathological form and a transfigured dynamism through therapeutic processes. To overcome this limitation, it is crucial to consider the notions of anthropological proportions and dialectics. The concept of anthropological proportion, coined by Binswanger (1956), alludes to the reference point to which the concrete human being can deviate or become disproportionate, constituting pathological behavior and typologies, even within a non-pathological domain (Kraus, 2001). Any psychic experience can be understood as the expression of relative proportions and tensions between opposite poles of the fundamental determinants within a particular individual’s life-world. A classic example provided by Binswanger was the spatial disproportion between verticality (height) over horizontality (depth) observed in schizoid personalities and schizophrenia. In these cases, patients experienced reduced or lost insertion in the everyday world and got stuck in a particular extravagant (delusional) ideal (Binswanger, 1956). There are many other proportions, such as distance vs. proximity, singularity vs. plurality, self-identity vs. role identity. Inspired by Jaspers (1913/1997) postulate that dialectics are the basis for understanding the immanent mobility of existence, Blankenburg (1982) has integrated dialectics into the study of anthropological proportions, allowing for a more dynamic approach to comprehend the progression and compensations of a particular condition. The global configuration of many (dis)proportions between different polarities can reveal a distinctive whole referred to as the “existential type” (Kraus, 1991). For example, *typus melancholicus* is characterized by the following disproportions: hyper-identification with roles, a tendency to imprison in past time (conservative), a strong attachment to orderliness, habituality, predictability, and stability. If these disproportions increase, a melancholic transformation occurs (Tellenbach, 1961/1975).

Although it may seem paradoxical at first to combine the identification of essence by classical phenomenology with dialectics—especially in its Hegelian sense of a back-and-forth process designed to combine opposites without elimination or reduction—this procedure is fruitful and has since been consolidated in what is known as dialectical essentialism (Messas et al., 2017; Messas and Tamelini, 2018). In the dialectic form of essentialism, the researcher works with a bifocal lens, focusing on recognizing the essential core of the psychopathology while observing the movements around which this core is transformed (Messas, 2021). The psychopathologist explores imbalances and identifies the components of opposites that become fixed or overdominant, constituting a typical anthropological disproportion and the dynamics of its transformation (Messas et al., 2022). In addition, the psychopathologist analyzes the compensatory movements^2^ to rebalance the disproportions, either from within or from without, induced by treatments. The essential core or typical style of transformation of existence should not be understood as a causal explanation but as immanent conditions of the possibility of specific experiences (Tatossian, 2002).

**Figure 1:**
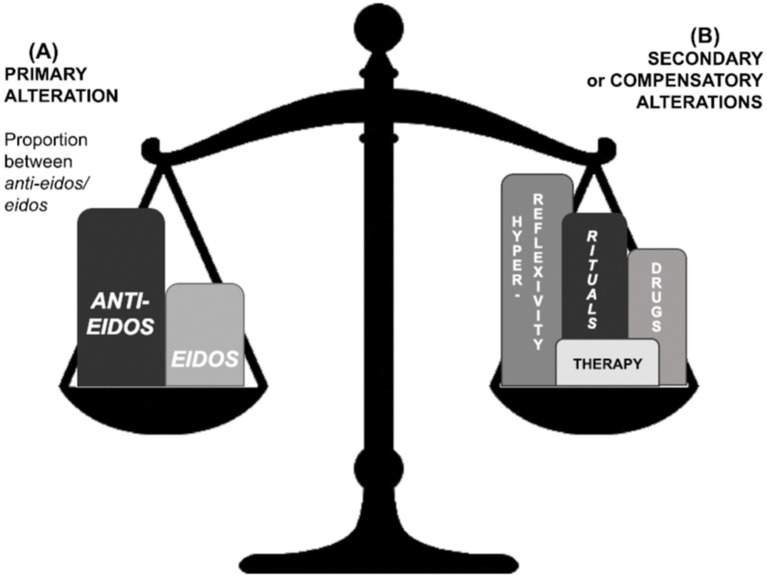
Relations of balance between primary **(A)** and compensatory **(B)** alterations in the obsessive–compulsive existential type. Primary alteration **(A)** essentially corresponds to the proportional relation between the *eidos* and *anti-eidos*. Compensatory alterations are structural rearrangements from within (such as rituals and hyper-reflexivity) or from without (triggered by medicine or psychotherapy).

Dialectical phenomenological psychopathology may be considered a suitable approach for studying dynamic obsessive–compulsive clinical presentation. The kinetic obsessive–compulsive psychopathological framework—as historically highlighted by Jaspers (1913/1997) and Binder (1936/1979)—is constituted by the interplay between two fundamental aspects or poles: the primary alteration (Figure 1A) and the compensatory mechanisms (Figure 1B) that is established to limit the imposition of essential core but may become counter-productive. The clinical pictures result from the combination of both aspects. Many symptomatic presentations come from dynamic changes, i.e., tendencies toward the predominance of one pole over the other and compensatory movements to reinstate balance between them.

Our main objective in this article is to provide a detailed analysis of the anthropological disproportions that characterize the obsessive–compulsive existential type within each fundamental phenomenological category: temporality, spatiality, identity, intersubjectivity, and corporeity^3^ (section 3). By understanding these disproportions, we aim to shed light on the compensatory structural movements that manifest as hyperreflexivity and rituals (sections 3 and 4). Furthermore, we intend to propose research and therapeutic interventions based on this understanding (section 5).

## 3. Dialectics of anthropological proportions

### 3.1. Obsessive–compulsive existential type: the supremacy of the *anti-eidos* over the *eidos*

Our work commences with the clinical intuition of the classical phenomenological works on obsessions by Gebsattel (1938/1966) and Straus (1938/1971) as a specific way of being in the world. Both authors identify a negative transformation of the life-world of the obsessive–compulsive person, in which the natural and harmless characteristics of the world are overshadowed by a predominance of disgust, decomposition, and death (Esposito and Goretti, 2022). Gebsattel (1938/1966) stated that the essential core of the obsessive life-world is the “lordship” of the “endogenous emergence of the *anti-eidos*,” which “makes vital sense, union, fusion, and contact impossible” (p. 124). For the German author, *anti-eidos* is a concept that “resumes all the diluting forces of existence” (Gebsattel, 1938/1966, p. 166). The intuitive grasp is further expressed in his work vaguely as “repulsive or annihilating power,” “primordial anchorage of disgust,” or “enemy force.” A dialectic intuition between *eidos* and *anti-eidos* can be traced from this work, which is enlarged upon in our study in the context of the obsessive–compulsive existential type.

**Figure 2:**
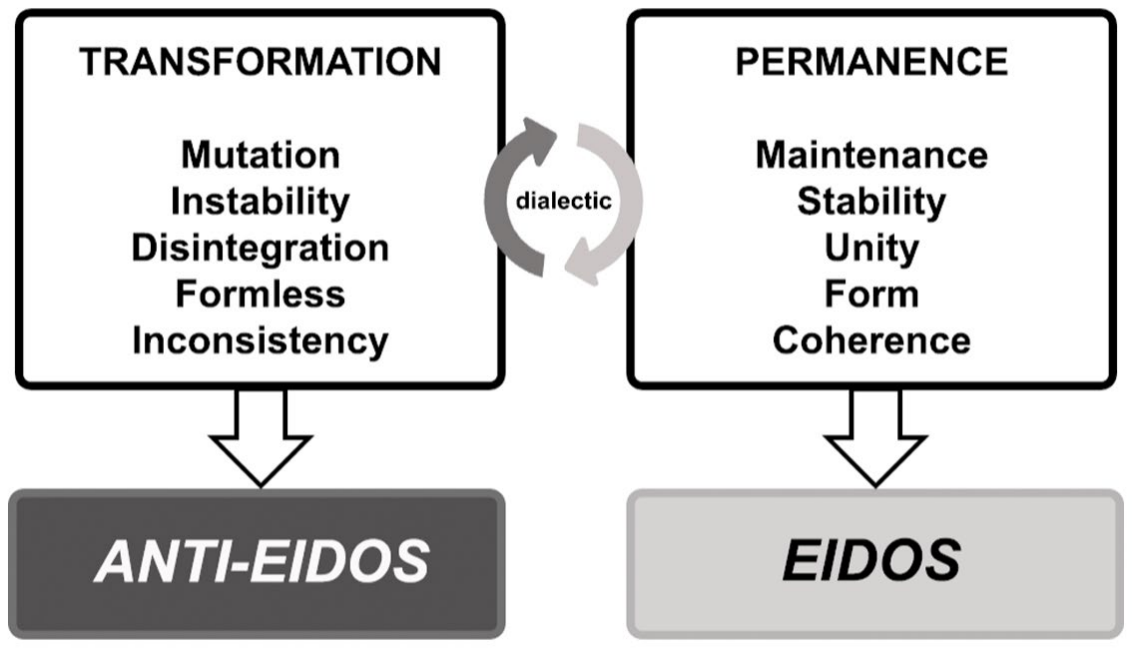
Dialectic between *eidos* and *anti-eidos* in human existence.

The continuous dialectical movement of permanence and transformation is elementary and inherent to all phenomena of nature, including human existence (Figure 2). Grounded on the work of Gebsattel (1938/1966), the force, which enables transformation, mutation, inconsistency, and disintegration, is named the anti-eidetic force, or *anti-eidos*. The opposing force that guarantees the maintenance, stability, unity, form, and coherence of existence is referred to here as the eidetic force, or simply *eidos* meaning the capacity mental life has to produce forms of existence (*eidos* = form in Ancient Greek). The latter ensures the fusion of being with the power of becoming, enabling well-being and naturalness. The former represents the diluting forces of existence that result in fragmentation, annihilation, threatening form, and distorting the connection of the self with becoming. At first sight, one might conclude that the *anti-eidos* is harmful and undesirable, yet this ensures that any structure remains incomplete and open to re-creation and renovation. The dialectic with the *eidos* prevents this openness from becoming excessive or chaotic, leading to nothingness.

**Figure 3:**
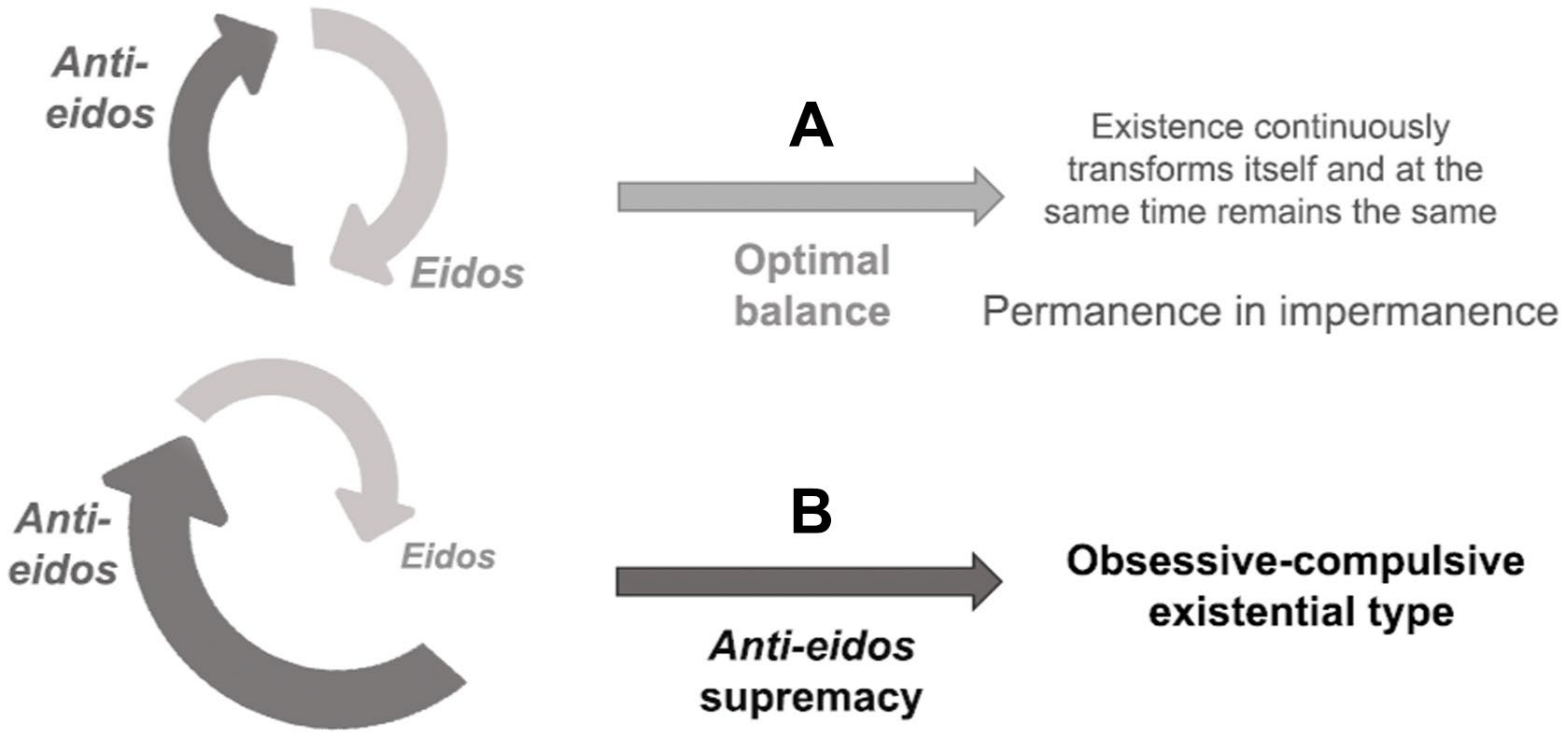
Proportion between *eidos* and *anti-eidos*: **(A)** optimal balance and **(B)** the supremacy of *anti-eidos* in the obsessive–compulsive existential type.

In normal conditions, there is an optimal balance (Figure 3A) in the dialectic between *eidos* and *anti-eidos*, and existence can flourish authentically. This homeostasis assures a fluid and future-directed (protentive) temporality, appropriate adjustment of the distance between the self and the world or the other, self-delimitation, self-agency, adequate embodiment, synchronicity, naturalness, and familiarity with the world or alterity. However, when the *anti-eidos* overpowers the *eidos* (Figure 3B), there are anthropological disproportions in the domains of temporality, spatiality, identity, intersubjectivity, and corporeity, both qualitatively and quantitatively, which will be analyzed below. The essence of obsession corresponds to the supremacy of *anti-eidos* over *eidos*. The greater the ratio of the anti-eidetic to the eidetic power, the greater the severity of the psychopathological condition, resulting in different clinical presentations within an individual patient over time and across patients.

### 3.2. Polymorphic temporality

In the obsessive–compulsive existential type, temporality^4^ is characterized by a decrease in flow and may even culminate in stagnation. This deceleration brings to mind the physics of pendula with different moments: loss of propulsion, transitory paralysis, resumption of movement in the opposite direction, and stagnation.^5^ All these moments are interconnected, and the division proposed here is only for didactic clarity.

The pendulum’s actions depend on its properties and the characteristics of the medium in which it is situated. In parallel, obsessive–compulsive temporality relies not only on the patient’s structure, vulnerabilities, and compensations but also on tractions and resistances from the situation in which the patient is embedded (e.g., the influence of the family and social contexts). Nevertheless, it is worth noting that individual risk factors seem more dominant than environmental ones in OCD (Fontenelle and Hasler, 2008).

In the works of classical phenomenological psychopathology by Straus and Gebsattel, temporality is identified as an appropriate category for elucidating the phenomena associated with obsessive–compulsive behaviors. Just as the slowdown and stagnation of a river lead to sedimentation and the proliferation of microorganisms, the loss of temporal flow in consciousness predisposes disgust, degradation, and putrefaction (Gebsattel, 1938/1966; Straus, 1938/1971).

#### 3.2.1. Loss of propulsion (deceleration)

**Figure 4:**
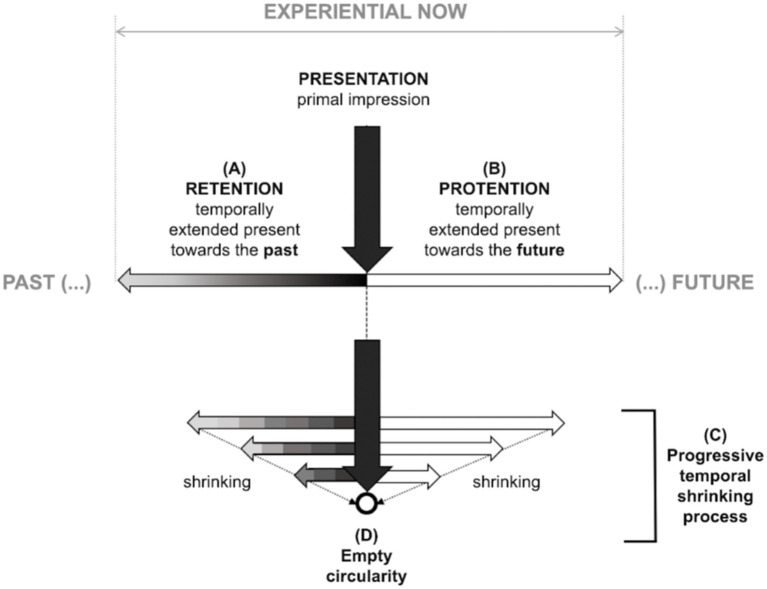
Temporal shrinking in the obsessive–compulsive existential type. The temporal structure comprises presentation, retention **(A)**, and protention **(B)**. The gray gradient on the retentive arrow **(A)** represents the imprint of a primal impression on retention. The protentive arrow **(B)** is empty, expressing expectation or intention to be fulfilled. In the obsessive–compulsive anthropological condition, a process of temporal shrinking **(C)** can culminate in empty circularity **(D)**. The shortened retentive arrows are represented by gray rectangles (in contrast to the gray gradient) to mark the lack of temporal fluidity.

The obsessive–compulsive temporal structure is not fragmented, as in the case of psychosis (Gebsattel, 1938/1966; Fuchs, 2013a), but it is not natural, future-directed, or continuous, either. It is prevented from flowing by a preponderance of anti-eidetic power, which compromises intentionality^6^—a vector associated with directness and orientation. Faded intentionality allows some consciousness units (as thoughts or images) to pop up from the tacit continuous flow of experiences. During the deceleration process, there is a change in the temporal structure (Figure 4), composed of presentation (primal impression), retention (extension of the present toward the past; Figure 4A), and protention (extension of the present toward the future; Figure 4B). The temporal structure shrinks: immediate retention takes precedence, and future-directed projections tend to be retracted (Figure 4C). This temporal constriction characteristic of obsessive–compulsive patients distorts the relation of intention with action. We can mention three clinical situations of this structural shortening. Firstly, patients may report imagined acts as if they were a *fait accompli* (thought-action-fusion; Shafran and Rachman, 2004). For example, they may experience the mere thought of committing a crime as having engaged in a criminal act. Secondly, patients tend to focus on the immediate relief of uncomfortable symptoms and often overlook the long-term consequences of their disorder (Kalanthroff et al., 2017). Thirdly, patients’ temporal constriction may mean they experience a remote possibility, such as death, with great proximity and without its transcendental quality.

The sense of agency refers to the experience of being the author or initiator of one’s actions or thoughts and is closely connected to self-awareness and intentionality (Fuchs, 2007a; Gallagher, 2021). Clinically, the debilitation of this pre-reflexive dirigibility expresses itself as diminished self-agency with consequences for the obsessive–compulsive identity (as will be shown in section 3.4).

#### 3.2.2. Transitory paralysis (empty circularity)

As retention and protention progressively shrink (Figure 4C), temporality becomes increasingly constricted, potentially culminating in empty circularity (Figure 4D). Intrinsic to the protention of any act of volition is an expectation or intention to be fulfilled (represented in Figure 4B as an empty arrow). Volition is not what triggers the act but the principle that permeates every step in the overall action (Spano, 2022). The partial steps accomplishments, usually transparent in our lived experience,^7^ become salient to the consciousness of obsessive–compulsive patients.

The obsessive–compulsive patient who hyper-focuses on parts and details risks overlooking the whole context of a situation. Then, the fulfillment of the overall act will not occur (Janet, 1903; Rovaletti, 2005; Zor et al., 2011) since there is a disarticulation between the actual action and the total temporal span, resulting in non-vectorial, infinitely repetitive circular time. For example, patients may partition the simple task of washing their hands into numerous isolated steps, such as how and how often they clean each finger. If they suspect they have made a mistake in any such movement, they feel compelled to start again from scratch and retrace their steps. They may even have trouble completing such a task. Clinical patients go into cycles of repetition and feel incomplete (Ecker and Gönner, 2006; Lee and Wu, 2019) and constantly uncertain (Stern et al., 2013). There is no possibility of transcendence in this circular temporality and obsessive–compulsive patients remain constrained, compressed, and fixed in a single mode of being.

#### 3.2.3. Resumption of movement in the opposite direction

When temporal fluidity is healthy, what was initially presented or experienced in one moment becomes retention in the next. Each moment builds upon the previous moment to create a continuous and cohesive experience of time (represented in Figure 4A by the gray gradient arrow). In the obsessive–compulsive existential type, as the enchainment of events becomes increasingly frayed, an artificial compensatory effort to maintain the juxtaposed instants from moment to moment (repetitive performances or behaviors) occurs to reconnect them to the temporal totality. The resulting non-volitional counter-movements are designed to force the juxtaposition of each step (represented in Figure 4C retentive arrows by the juxtaposition of gray rectangles). Still, they are unnatural, lacking in fluidity, and may fail to reconnect each action to task^8^ (Gebsattel, 1938/1966). Such compensatory measures, while attempting to resume fluidity, dirigibility, and continuity, paradoxically often end up in an even more significant weakening of intentionality. As the individual fails to regain control, they start to doubt themself even more (De Haan et al., 2013b). This self-destructive cycle can culminate in pervasive slowness or exhaustion.

#### 3.2.4. Stagnation

There are certain patients who are unable to uphold their compensatory processes, which can lead them to become exhausted. This is like how the immune system functions when it tries to control a foreign substance or illness. The immune system may react in various ways, such as forming a granuloma (an empty circle) to contain an infection or causing a cytokine storm (a systemic inflammatory response). This can result in unmanageable bodily dysfunction (failure of defense/consumption) which can be difficult to handle. Intentionality is nullified, and self-agency impairment can resemble that of a psychotic schizophrenic patient. Despite the same clinical presentation, the structural configuration is distinct.^9^ In this state, the obsessive–compulsive patient can be classified as having poor or absent insight. It is worth mentioning that many studies discuss the possibility of a continuum or differential diagnosis between obsession and psychosis (Bürgy, 2007; Oulis et al., 2013; Palermo et al., 2020; Rasmussen et al., 2020). Let us see an example of insight modification:

“*When I began experiencing obsessive aggressive thoughts of harming my colleagues with a knife*, *as well as blasphemous thoughts during religious masses*, *I was acutely aware of how absurd and inappropriate they were*. *Coming from a devout Catholic family*, *such thoughts and actions were completely unacceptable to us*. *I fought relentlessly against these intrusive thoughts*, *attempting to neutralize them through prayers or acts of kindness*. *This struggle has persisted for over twenty years*. *However*, *for about six months*, *I felt utterly drained*. *People say I accept them now*. *I am unsure if these thoughts are mine*, *but I no longer care*” Clinical impression: patient shows indifference. Obsessive thoughts no longer have the same meaning or power over him. He has isolated himself from social interactions and withdrawn from work and religious activities [Patient A].

Longitudinally, experiences are not sedimented into a narrative unity. In the extreme, impaired temporalization (Gebsattel, 1938/1966) or arrested immanent temporality (Dörr-Zegers, 2018) results in a compromised quality of historical time (Straus, 1938/1971). The impossibility of achieving authentic historicization or the obstruction of becoming is associated with the obstruction of self-actualization (De Haan et al., 2013a), which feeds back negatively to intentionality itself, causing existence to become more rigid, confined, and static (Rovaletti, 2005).

### 3.3. Spatiality: no calibration of distance

Spatial disproportion results from the accentuation or absolutization of one pole. Some spatial poles described in phenomenological psychopathology are “proximity–distance, horizontality–verticality, centrality–peripherality, and centripetal–centrifugal” (Messas, 2021, p. 23). Classical (Gebsattel, 1938/1966; Straus, 1938/1971) and contemporary (Rovaletti, 2005; Dörr-Zegers, 2018) phenomenological psychopathology have emphasized the importance of proximity–distance in the life-world of the obsessive–compulsive patient, observing a deterioration in the patient’s ability to calibrate these two poles (Rovaletti, 2005; Dörr-Zegers, 2018).

Lived distance refers to the lived space that connects people to their environment (*gestimmten Raum*; Binswanger, 1933/2006) and their horizons of possibilities (Fuchs, 2007b). The two opposite ends of the proximity-distance spectrum are fusion and disconnection. In these areas, it is impossible to truly recognize the world or alterity, as the self either merges with the world or infinite spatial indeterminacy occurs. If a balanced distance from reality is not achieved, one’s relationship with oneself, objects, and others deteriorate, and space loses its coherence (Tellenbach, 1983; Dörr-Zegers, 2018). Patients with obsessive–compulsive disorder are unable to feel a sense of familiarity with the world, others, or their own experiences.

Obsession derives from the Latin *obsedere*, surrounding or enclosing something or someone, besieging, fencing in, blocking. Just as temporality is restricted, the spatiality of the obsessive–compulsive patient is characterized by constriction, oppression, limitation, and narrowing. Patients increasingly withdraw into a limited space, progressing to social isolation (Timpano et al., 2014), confined to the corner of a room, or stuck in their bodies (detailed in 3.6). The phenomenological compensation for this anthropological spatial disproportion is designed to reinstate distance. This can occur through avoidance or rituals (described in 3.6 and 4). We include an excerpt from a letter written by a patient of Straus to illustrate the spatial disproportion and the structural effect of compensatory ritual:

*“If one of these persons touches me*, *I must clean or wash the cloth*, *either with soap or benzene*. *If some of these people enter our home*, *I cannot move anymore*. *I feel the room would get narrower*, *and I could not avoid touching everything with my dress*. *I have to go through the door edgeways*. *To find peace again*, *I must wash everything with soap and water*. *Then everything becomes large and wide*, *and I can move again*” (Straus, 1948/2016, p.61).

Constriction and narrowness also distort the patient’s affectivity and meaningful disclosure. In other words, temporal and spatial constrictions contribute to the affective atmosphere of disgust and repugnance that colors all obsessive relations (in terms of attunement with the world, with others, and with one’s horizon of possibilities) and modify the patient’s sense-making.

#### 3.3.1. Affectivity

The predominance of the *anti-eidos*—with spatial constriction associated with a slowing down of temporal fluidity—allows the emergence of a “pathic” structural backdrop^10^ of disgust, repugnance, impurity, decay, and decadence, as classically described by Gebsattel (1938/1966) and Straus (1938/1971) and corroborated in recent findings (Bhikram et al., 2017). Obsessive–compulsive patients are overwhelmed by this baseline mood, manifesting anxiety, distress, and fear and reacting compensatorily with repulsion, aversion, and avoidance to impose distance. This affective atmosphere has an elusive yet ubiquitous quality that can pervade the patients, their lived space, and their interpersonal relations (Fuchs, 2013b; Brencio, 2018). Depending on how much this atmosphere takes over the patient, they can present with extremely diverse clinical manifestations. If the anti-eidetic force emerges sporadically or is circumscribed to specific sectors of life, the very fluidity of existential becoming can dissipate the atmosphere of disgust. Transience is an integral part of any atmosphere, but it tends to be disfigured in the obsessive–compulsive existential type by permanence and non-evanescence. Disgust and repugnance do not dissipate. While some patients observe daily rituals that cause little harm, others can become absorbed by these rituals to exhaustion. We present a clinical vignette that portrays the ubiquity of disgust spreading throughout the patient’s experiences:

“*I began experiencing marital difficulties with my wife*, *which led me to search for pornographic content online*. *Since then*, *I have started feeling dirty and have difficulty caring for my youngest daughter*. *I am spreading something bad everywhere I go*. *I need to wash my hands meticulously*, *focus on each finger*, *and repeat the washing process 13 times before I can contact my daughter*. *As a result*, *my hands have become so injured that they are bleeding*. *I constantly fear that my blood will contaminate every object in my house or public transportation*. *I am impure*. *I'm afraid I'm going crazy*. *This fear has caused me to distance myself from my daughter*, *and I feel an overwhelming sense of guilt for relinquishing my role as a parent*” [Patient B].

#### 3.3.2. Meaningful disclosure

Human beings are always situated in a mood that attunes them to the world and gives meaning pre-conceptually and pre-theoretically (Brencio, 2018). Affectivity renders the subject prone to perceive a situation, feel, interpret, and act in a certain way. Affectivity (feeling, “pathos”) and cognition (understanding, “gnosis”) interact in such a way that allows access to oneself, the world, and alterity (Ratnapalan and Reggio, 2012). The restricted horizon of possibilities and temporal constriction seen in the obsessive–compulsive existential type transform how patients establish relations of meanings (valence, affordance, relevance, and significance) that guide their intentional and nonintentional acts.

The influence of affectivity on meaning assignment can be symptomatically expressed as an overestimating threat, attentional bias to danger, sensitivity to feared stimuli, and anxiety (Kalanthroff et al., 2017). Obsessive–compulsive patients cannot change their restricted system of reference in the moment of perception as they cannot attribute meaning to themselves. The unpleasant, fearful, or immoral aspects of objects or situations gain salience and stand out from the whole, consuming the patient’s attention (Dörr-Zegers, 2018). For example, a patient may perceive a flower as associated with cemeteries and death, overshadowing any other association, like gentleness and kindness. It is, therefore, impossible to speak here of choice or decision on the part of the self when it comes to sense-making—a topic we will cover further in item 4. Clinically, this imposition can be manifested in experiences of relative strangeness or absurdity, coercion, or lack of control. In the vignette below, we can observe how crosses, flowers, or hospitals at the perceptual moment are immediately associated with death and tragedy by the patient without his conscious deliberation:

"*Ever since my uncle died in an accident*, *I feel compelled to walk in a specific way – I need to touch the ground with my heel and the entire lateral side of my foot at once*, *and the hallux pointing to the only church in town – to prevent another accident for happening to a loved one*. *If I don't proceed in this way*, *I become restless*. *Merely seeing a cross or a flower bouquet triggers immense discomfort*, *as they feel like forewarnings of disaster*. *Since my brother started working in a hospital*, *I haven’t been able to get close to him*; *he has become the personification of diseases and death in my mind*. *I know that is absurd*, *but I can't stop thinking or acting this way*. *Despite my feet and knees hurting*, *I can't stop the habit*. *This behavior has become time-consuming and uncomfortable*, *making even leaving home feel like a burdensome chore"* [Patient C].

In summary, the constituted temporal and spatial aspects of the obsessive–compulsive patient’s conditions of possibility can be accessed from the perspective of affective (emotional) or cognitive (constitutive) presentations. From these, it can be understood why historically, obsessive–compulsive condition has been considered a disorder either of emotion or intellect.^11^

### 3.4. Self-awareness and identity: integral self but with a fading sense of activity

According to Parnas and Zahavi (2002), self-awareness can be described at three concentric hierarchical levels: the nuclear layer or minimal self, self-awareness as an agent (or “I-consciousness,” which we call I-pole), and narrative self-awareness.

The minimal self is the condition of possibility of the other levels and occurs pre-reflexively, immediately, non-inferentially, a-thematically, and tacitly. It ensures all lived experiences. Unlike their psychotic counterparts (Fuchs, 2015), obsessive–compulsive patients have no fracture in this most fundamental hierarchical level, responsible for self-delimitation (Jaspers, 1913/1997) and the particular aspect of “mineness” (ownership) seen in all psychic manifestations (Cermolacce et al., 2007). The acts of psychic life come from an “I” revealed, for instance, in the patient’s understanding that it is “my” obsession. The differential diagnosis between OCD and psychosis can be traced back to this level.

The second level, which corresponds to self-awareness as the agent, source, center, and pole of intentional experience, is undermined in obsessive–compulsive patients. The sense of agency is a pre-reflective (first-order) experience and a reflective (second-order) cognitive process of judging (Gallagher, 2021). As described above (section 3.2), dwindled self-agency expresses the “disempowerment” of the intention vector. In the obsessive–compulsive existential type, there is an integral self, but it is fragile,^12^ which is to say it is characterized by the experience of non-dirigibility (Stanghellini and Ballerini, 1992) and depleted intentionality. Distortions of the sense of activity result in a weak awareness of acting and performing. This means that there is no experience of completion, consummation or realization (Dörr-Zegers, 2018). Clinically, obsessive–compulsive patients may experience incompleteness, lack of will control, and, in a more radical way, depersonalization and derealization (Janet, 1903; Bürgy, 2012; Tatlı et al., 2018). In the long term, there is no sedimentation of experiences into a narrative unity.

Identity stems from incorporating the temporal aspect into self-awareness. “I-consciousness” enables synchronic coherence (being particular, singularity), diachronic identity (being the same all the time, permanence), and unity of the stream of consciousness (Parnas and Zahavi, 2002). Identity is permanently built through the pre-reflexive preservation of a particular style. This involves an implicit comparison between what arises in the consciousness and the individual’s previous experiences (Messas, 2021). In other words, an individual will constantly but pre-reflectively check whether a lived experience is consistent with their vital flow. Subsequent experiences are assimilated or denied non-volitionally. As such, non-identity (or identity absence) also constitutes identity (Messas, 2021). In the obsessive–compulsive existential type, both are lived ambiguously. For example, a person may perceive a thought to be their own but regard it as irrational, strange, or incongruent to themself (Uvais and Sreeraj, 2017), as if it was being forced on them (Gebsattel, 1938/1966).

The last level of self-awareness, corresponding to personhood and narrative self-awareness, is the most sophisticated and complex. The notions of self-image, self-esteem, self-social, and personal identity are framed at this level (Parnas and Zahavi, 2002). Self-identity consolidates itself historically and emerges, mediated by language, in a narrative. A biography is toned by the cultural, social, family, interpersonal (relational), and economic context. Subjects adopt a combination of explicit and implicit values, settling on a hierarchy of principles of conduct and experiences by which they conduct their life (Fulford and Stanghellini, 2019). Obsessive–compulsive patients usually face dissonance between their lived experiences and values (more details in the section 4), distorting their self-image and self-esteem. For example, patient A reports a mismatch between his obsessive experience and religious beliefs and how the dissonance can undermine his relationship with himself.

The first two levels occur tacitly or pre-reflectively, for the most part, while the third operates mainly through an attentional, intentional process. Latent and reflective forms of self-awareness must work in equilibrium. In the obsessive–compulsive existential type, tacit phenomena can become disfigured due to self-agency fading. Compensatorily, reflective self-awareness becomes exacerbated and even turns the implicit aspect into the object of reflection to control the experiential transformation (Sass and Borda, 2015). The explicitness of tacit phenomena ultimately disrupts functionality (Fuchs, 2011; De Haan et al., 2013a). The manifestation of reflective self-consciousness in clinical cases depends not only on the temporal lag (*décalage*), but also on the ability to calibrate distance from one’s experiences (to switch perspective), ranging from the exacerbation (clinically expressed as increased recapitulation, rumination, and self-doubt) to the abolition of self-reflection (clinically defined as poor or absent insight). Obsessive–compulsive patients with intact insight faculty present various degrees of compensatory hyperreflexivity (Sass, 2010) or hyper-rationalization (Esposito and Goretti, 2022).

The preservation of the reflective capacity in obsession is stressed in the terms: *folie lucide* (Jaspers), *manie sans délire* (Pinel) and *folie avec conscience* (Baillarger). Too much reflectivity and control can culminate in a lack of freedom paradoxically (De Haan et al., 2015). Obsessive–compulsive patients have difficulty relaxing their control system (Kalanthroff et al., 2017), which can become the self-destruction principle (Gebsattel, 1938/1966). Then, their self-image is experienced as insufficient, impotent, inadequate, and unacceptable, which can impair the intentional vector further. To counterbalance their self-doubt (Godwin et al., 2020), remove any uncertainty,^13^ and regain a sense of control, they may engage in rituals involving checking, ordering, symmetry, exactness, perfectionism, and scrupulosity (Bürgy, 2019).

In the obsessive–compulsive anthropological framework, the modification at the third level also affects the reflection of the self as a historical and social being belonging to a broader context. Identity is found and functions in close relation to intersubjectivity. Kraus (2016) and Dörr-Zegers (2008) present the dialectic between self-identity (being-for-oneself, self-determination) and role identity (being-for-the-other, assumed identity based on social function). This dialectic is jeopardized in obsessive–compulsive patients. They show no elasticity among role identities, and their social and family roles are neglected. Inauthentically, autonomy is undermined and self-identity may become perilously attached solely to the role of the mentally ill patient (Pedley et al., 2019).

Let us illustrate with a clinical vignette:

*"I have always been an insecure child*, *and despite growing older*, *my insecurities have worsened*. *My mind is invaded by images of my home destroyed*, *burgled*, *or burnt down*. *I try hard not to think that way but I can't avoid it*. *I constantly fear leaving the doors or windows open*, *worrying that it will result in a burglary*. *I'm also afraid of forgetting to turn on the stove*, *which may lead to a tragic event*. *To alleviate these fears*, *I check the locks at least five times*, *even with my wife supervising me*. *My wife demands that I stop the checks*, *but I can't avoid doing them because it increases my distress and anxiety*. *I am hostage to my thoughts*. *At work*, *my doubts affect my performance*. *I worry about putting obscene words in the middle of important reports or emails*. *I know I'm decent and ethical but don't trust myself*. *I frequently miss assignment deadlines because I obsessively reread documents word for word*. *I can't finish a task*. *I need the reassurance of a colleague or my boss for proofreading*. *Unfortunately*, *my last evaluation yielded negative feedback*, *putting my job at risk*. *Since I started getting stuck with symmetrical arrangements at home until I felt they were just right*, *my wife has grown tired of these problems and is considering divorce*. *I'm an absolute disgrace*. *I am the incarnation of shame*. *I want to improve*, *but I see no way out"* [Patient D].

### 3.5. Intersubjectivity and interpersonality: close proximity between self-pole and other-pole

Interpersonality corresponds to the manifestation of existence and its implantation in the world of equals. It is how an individual relates to their peers. What underlies and determines each personal and interpersonal experience is intersubjectivity (Messas, 2021). Intersubjectivity is the condition of the possibility of subjectivity and all encounters. Intersubjectivity is constitutive and simultaneously constituted by subjectivity. One relational dialectic is the proximity-distance between self-pole and other-pole. In the obsessive–compulsive existential type, the self-pole is very close to the other-pole, which impairs the recognition of the others and then metamorphosizes their physiognomy. Other is tainted by the baseline atmosphere of decay (as described in section 3.3) and not appears as a constituted historical person. It is not possible to keep a safe distance from alterity; others cease to be harmless, innocuous, understanding, or natural. Some patients report ambiguous and contradictory feelings and cannot deal with negative emotions (Cludius et al., 2021). For example, a puerperal patient experienced difficulty understanding intrusive thoughts of strangling or hurting her beloved newborn baby with a knife. She required constant support and companionship for a year and had to take precautions by securely locking all the knives inside a cabinet.

Unlike psychotic patients, obsessive–compulsive ones maintain a preserved implicit pre-reflexive other, ensuring the co-constitution of reality. In other words, in the obsessive–compulsive existential type, there is no intersubjective default but rather an imbalance between self and other poles. Consequently, obsessive–compulsive patients show mild disturbances of common sense, while schizophrenic ones seem to truly abdicate it, losing natural self-evidence (Blankenburg, 2001). The intersubjective proximity empowers the experience of embarrassment and shame^14^ (Weingarden and Renshaw, 2015), hindering the identification of obsessive–compulsive experiences as problematic. The experience of stigma and shame means that diagnostic and therapeutic help is often sought at a late stage (Glazier et al., 2015). As early treatment is associated with a more favorable prognosis, this late onset of treatment is a factor that contributes to the exacerbation of the condition (Fineberg et al., 2019).

According to Straus (1948/2016), obsessive–compulsive patients experience a crisis of coexistence. They tend to isolate themselves and create a self-imposed state of siege, impeding their ability to engage in meaningful interpersonal relationships. Obsessive–compulsive patients live in isolation (Kraus and Csef, 1995), inside themselves (Dörr-Zegers, 2008), in a heavily armed fortress with all accesses blocked and controlled (Stanghellini and Ballerini, 1992). The movement to self-isolate from others maintains and reinforces the crisis of coexistence. Francesetti (2017) describes obsessive–compulsive patients as living in the solitude of terror, seeking relief in the distance. Some patients can avoid interpersonal contact by doubting their self-regulation because they fear harming others, saying wrong things, having unwanted impulses (killing or sexual desire), and potentially acting on these impulses. In severe cases, intersubjective communication can be restricted to close caregivers, frequently only to perpetuate rituals. The accommodation phenomenon occurs when the family participates directly in the rituals or actively avoids symptom triggers (Renshaw et al., 2012). The clinical vignette below exemplifies accommodation:

*“Once*, *while watching a TV program about leprosy at my grandmother's house*, *I became obsessed with falling ill and becoming disfigured*. *Consequently*, *I began avoiding contact with people*, *carrying disinfectant everywhere*, *avoiding public bathrooms*, *refraining from eating outside my home*, *and exclusively wearing pants and long-sleeved shirts*, *even during the summer*. *Every time I return home*, *I engage in a ritual that lasts nearly three hours*, *showering and meticulously tending to my clothes*. *Due to excessive absences and the inability to participate in group work*, *I lost an entire school year*. *My parents wake up three hours earlier than their appointments to assist me with my morning routine*. *They have also stopped visiting relatives' houses to avoid the responsibility of caring for their clothes and shoes upon returning*, *and they bear the burden of quintupled water consumption in our household*. *They chose to change their lives rather than deal with my lack of control*" [Patient E].

The greater a patient’s impairment, the more difficult it is for them to perform their role identities properly. In such cases, they can barely be distinguished from their mental condition, and it becomes unsustainable for them to perform their role in a family (Pedley et al., 2017; Muhlbauer et al., 2021) or hold down a job (Mancebo et al., 2008).

### 3.6. Corporeity: disgust and repulsion—protecting own boundaries

The anti-eidetic threat of annihilation or dissolution of form has repercussions on corporeity. Experiences of extreme proximity (described in the section 3.3) are a source of concern, internal somatosensory sensations (Bragdon et al., 2021), or visceral discomfort that border on the psychotic. Decomposition, putrefaction, disintegration, liquefaction, viscosity, loss of form, adherence, and return to matter are associated with this experience of loss of contiguity. The compensatory movement exacerbates surveillance of the organism’s frontiers to re-establish distance and prevent contact, protecting the self through avoidance, expulsion, or repulsion (as shown in patients B and E). Thus, the obsessive–compulsive patient lives in bodily tension and spends much energy maintaining their boundaries.

According to evolutionary science, disgust is considered an adaptive response of human nature and is expressed ubiquitously and universally (Korsmeyer, 2009), albeit mediated by cultural variables. It is a robust bodily emotion—a protection of integrity—like fear. Its function is to avoid contamination, contagion, infection, and poisoning. Some authors regard disgust as the psychic correlate of nausea or vomiting^15^ in the physical body. According to Strohminger (2014), it is an extension of the immune system in defense of the organism.

Repugnance is essentially tied to sensory impressions and therefore remains primarily inaccessible to knowledge, deliberation, or rationalization (Fussi, 2018). The sensory systems most associated with proximity and immediate dominance are taste, smell, and touch (Tellenbach, 1985). It is a common clinical observation that obsessive–compulsive individuals may experience preoccupation or discomfort when it comes to being touched or touching other people or objects. They avoid greeting, kissing, and hugging. Experiences of unpleasant smells from their own body, which patients fear are affecting others, also express this dissolution of form.^16^ The patient can have repulsion toward one’s own body and can self-isolate. Excessive concern about becoming pregnant or having diseases can also express a fear of breaking frontiers. A safe distance can be maintained or re-established by avoiding contact or engaging in more rigorous washing, cleansing, or decontamination rituals (Bürgy, 2019).

It is worth pointing out that not only the physical body is threatened by “formlessness.” The lived body also includes another delimitation corresponding to dignity, honor, or moral value. In addition, it is common for obsessive–compulsive patients to experience degradation, depreciation, discredit, or indignity, with a concomitant need to defend themselves from sexual, aggressive, or immoral thoughts and acts. The patients can fear blurting out obscenities, blasphemy, or insults. Blurred boundaries can lead to the impression that intimate contents can overflow into the external space. For example, patient B believed that his pornographic thoughts could contaminate his daughter if he touched her without first washing himself. Obsessive–compulsive patients may attempt to protect themself with mental processes to keep away undesirable ideas, magical beliefs to prevent adverse outcomes and prayer rituals. As the condition worsens, individuals tend to develop a rigid routine and limited scope of action (Rovaletti, 2005). We illustrate with a clinical vignette:

"*I started going to the gym because I felt very skinny*. *I soon realized that I was paying too much attention to the size of other men's genitals*. *I tried to avoid such thoughts*, *but I couldn't stop them entirely*. *These thoughts made me question my sexual orientation*. *Am I might be gay? If someone approaches me to talk*, *I can't maintain eye contact and avoid shaking hands*. *I started avoiding being alone in the locker room with these men*, *fearing that I might lose control and sexually assault them*. *I only shower in my house*. *When these thoughts arise*, *I repeat seven times that I like women and ask God for forgiveness fourteen times*" [Patient F].

Indeed, the significant correlation of OCD with tic disorders and Tourette’s syndrome is related to the bodily aspect. This disorder is characterized by motor and vocal tics (i.e., rapid, repetitive, stereotyped movements, or vocalizations; Leckman et al., 2006). Some studies suggest that patients with OCD associated with tic disorders tend to have an early onset and more severe symptoms. Phenomenological research on Tourette’s syndrome (Curtis-Wendlandt and Reynolds, 2021) points to a correlation with a sense of (dis)ownership or (dis)agency of embodied actions, loss of habitual confidence, and a battle against one’s own body’s uncontrollable movements. A parallel between motor and psychic autonomization can deliver valuable hints about the links of obsessive–compulsive conditions with biological substrates.

## 4. Phenomenological compensations—failure and clinical features of psychic autoimmunity

Permanence and transformation have intrinsic ambiguities, i.e., both have positive and negative facets and should operate in an optimal balance. Unlimited change can culminate in destruction and chaos, while too much permanence can stifle existence. The existential structure has a proper regulatory system to maintain homeostasis between this fundamental dialectic. We propose an analogy of this system to the autoimmune system, given that its purpose is to defend^17^ and rebalance the structure. Phenomenological compensations correspond to structural rearrangements from within the existential structure (in each of the fundamental phenomenological categories and combinations thereof). In addition, we point out that therapeutic practices can induce compensation from without.

**Figure 5:**
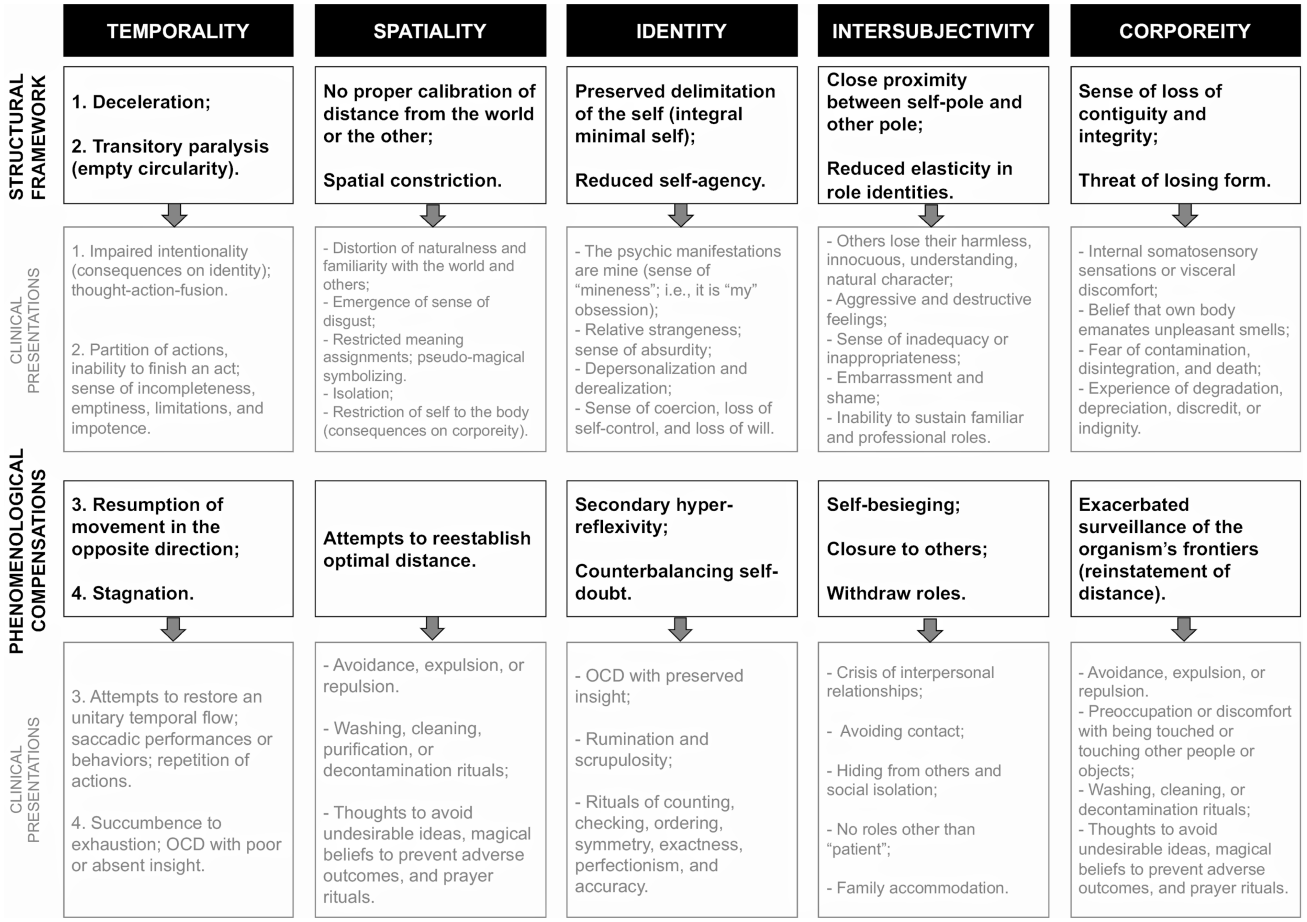
Effects of anti-eidos supremacy over the *eidos* and the structural compensations on temporality, spatiality, identity, intersubjectivity, and corporeity. We correlate structural configurations to clinical manifestations.

When there is an anthropological disproportion, the existential structure naturally readjusts itself. As we exposed the main disproportions existing in the obsessive–compulsive existential type in section 3, we also briefly presented the compensatory movements (summarized in Figure 5). In the obsessive–compulsive existential type, the temporal and spatial disproportions are, respectively, the decelerated temporal flow and the predominance of proximity over distance. The global structure reorganizes itself, forcing temporal fluidity conservation to resume intentionality, preserving self-agency, and imposing reinstatement of the optimal distance between the self and the world/other.

As long as the autoimmune system works appropriately, it is unlikely to be noticed. To the extent that it runs into disruption, it becomes evident to us. Autoimmune diseases are the cost of having a defensive system. If compensations work ordinarily, the disturbance can be restricted to just one field of the patient’s life. However, compensation can be so dominant that it leads to a failure of the defense process. The exacerbation or disruption of structural compensation determines specific clinical pictures in the obsessive–compulsive patients, such as compulsive rituals and obsessional doubts. Compensatory strategies can evolve into more destructive counter-productive effects that trigger an inextinguishable dysregulated cascade, which worsens the prognosis,^18^ either with too much intensity or biased attacks on specific targets. For example, the primary sense of uncleanliness experienced by a patient does not possess the mundane solidity of width, height, and depth (Gebsattel, 1938/1966); the dirt and impurity are not material but symbolic. Their actions are designed to override or contain an anti-eidetic effect but are restricted to the superficiality or materiality of the praxis. As such, mere mechanical washing or cleansing rituals will never serve to eliminate the impurities and must be repeated incessantly. Thus, designed to regain direction, these mechanisms are doomed to fail from the very outset sysifically.^19^

Checking, reviewing, and recapitulating to achieve correctness, precision, exactness, counting, cleanliness, and preservation are all proper actions of all human beings in non-pathological scenarios. Obsessive–compulsive symptoms can be comprehended as the negative or uncontrolled face of the vital capacity of self-adjustment and self-protection inherent and constitutive to all human beings. What occurs in the obsessive–compulsive existential type is a quantitative increase in these actions (e.g., extreme perfectionism and exaggerated collection) and their qualitative distortion. A healthy person always maintains a degree of transcendence, some openness to incompleteness, imperfection, provisional, uncertainty, and unfinishedness, making evolution irrevocable. Uncertainties are unavoidable because all conquests must be updated and reasserted continuously. Figuratively, while in an obsessive–compulsive patient, repetition assumes a closed circular form, in a healthy individual it becomes an evolutionary spiral, maintaining a future-directed (protentive) direction and inhabiting a vast and uncircumscribed horizon.

Self-assessment, doubt, and control deserve further detail due to their relevance in clinical presentations in the obsessive–compulsive existential type. According to Wittgenstein (1969), doubts presuppose a background of certainties. At the most fundamental level, there are certain lived certainties that we do not question and take for granted in our relationship with our surrounding world and with others. These fundamental certainties shape what we refer to as common sense. Schizophrenic patients lose the natural evidence and may doubt what is obvious to others, as seen in Blankenburg’s patient Anne (Blankenburg, 1971/2013).

On the other hand, obsessional doubts seem to arise from a different level, as they do not abdicate from common sense rules. Having examined temporality and self-awareness, it has become evident that obsessive–compulsive patients experience intentional blunting, which hinders them from completing their actions. This lack of fullness impairs the sedimentation of their experiences and, consequently, prevents the development of confidence, habituality, and the sense of being rewarded. Longitudinally, they struggle to establish certainties about their actions. As a result, they may feel insecure, incomplete, and unreliable (e.g., patient D). They crave external or rigid sources of certainty, such as reinsurer person, recheck, numerical security, prayers, and taking extra care of their bodies against disease and contamination. These manifestations can be superficially interpreted as a need for control and intolerance of uncertainty.

Another human process of self-evaluation and self-correction involves the coherence of values, or, as we will describe here, the (in)consistency of the positional and value senses. An obsessive–compulsive person’s typical anthropological disproportions (outlined in the section 3) define an obsessive–compulsive positional sense as a source of meaning. This primal pre-reflexive structural architecture limits purposes continuously and spontaneously (even before they are expressed in consciousness) and delimits a restricted field in which freedom may act. Positional sense is given and is not a patient’s deliberate choice. The essence of the obsession arranges the existential structure, providing relevance to the world and endowing meaning in a narrow, unilateral, monotonous way (as described in the section 3.3). The themes that symbolize the loss of *eidos*—such as uncleanliness, contamination, putrefaction, death, aggressiveness, destructiveness, and immorality—prevail. In obsession, a group of objects—reciprocally connected by an ordinary meaning—stand out from all the other objects in the world (Dörr-Zegers, 2018). Clinically, obsessive–compulsive patients can choose neither their contents nor how they appear (e.g., patient C). As a result, they can experience this restriction as intrusion, coercion, lack of self-control, and loss of will.

The original pre-reflective form of the personality, which we call positional sense, dialogs dialectically with the more conscious assignment of the values to an experience by the same personality (value sense) and strives for harmony. The value sense is the first to be accessed and directly experienced by the patient (Messas, 2021). Each value sense is highly individual and corresponds to a hierarchy of principles of conduct and experiences by which patients guide their lives (Fulford and Stanghellini, 2019). The value sense incorporates temperamental tendencies, past biographical decisions, socioeconomic and cultural contexts, family customs, and intimate and significant interpersonal relationships. In the obsessive–compulsive existential type, a dissonance is observed between the positional and value senses (e.g., patient A and D). From this—and remembering Freud’s descriptions of obsessional neurosis—it is easy to see why obsessive–compulsive patients prone to hyper-responsibility or with firm conservative convictions, from repressive families not open to divergent opinions, or societies with too many taboos will tend to feel even more meaningless or incoherent.

The degree of dissonance between patients’ values and structural modifications (positional) can vary, configuring distinct impairments and severities. The greater the self-perceived incoherence, the greater the lived experience of anxiety, anguish, suffering, guilt, inadequacy, inappropriateness, embarrassment, shame, lack of control, and lack of freedom. This feature influences the self-perception of illness and treatment-seeking. Addressing this dissonance can be a therapeutic route toward reducing psychic suffering. How and to what extent disharmony is present in OCD cannot be overlooked, because it also helps the differential diagnosis and is essential in preparing therapeutic plans.

The mismatch also brings to mind the psychoanalytic term ego-dystonia. In classical psychopathology (Jaspers, 1913/1997; Schneider, 1959), the ego-dystonic aspect of obsession helps differentiate OCD from delusion, overvalued ideas, and anankastic personality. Oulis et al. (2013) highlighted that ego-dystonia relates to an incongruence between one’s concerns, thoughts, and actions and self-image. It is appropriate to use the term ego-syntonia in an anankastic personality,^20^ given that the obsessions are at the service of a perfectionist way of being. Conversely, in delusion, it would not make sense to speak of ego-dystonia—referring to dissonance—since it is a phenomenon of the pre-reflexive sphere, which precedes the moment of reflective self-awareness. In consonance with Oulis et al. (2013), our dialectical phenomenological description of the obsessive–compulsive patients’ life-world provides a more detailed view of the ego-dystonic phenomenon, contributing to a better differential diagnosis.

## 5. Future perspectives for scientific research and therapeutic proposals

Our enlargement of the phenomenological understanding of the obsessive–compulsive existential type helps build a sense of established therapeutic practices and raises other potential courses of action. Knowing which anthropological proportions are out of balance and how compensatory dynamics occur enables the condition to be categorized and staged more effectively. Also, it allows more effective decision-making regarding where and how to take pharmacological and psychotherapeutic actions to redress the miscalibration of proportions.

### 5.1. Refinement of diagnostic and severity measurement instruments for research and treatment

The operational diagnostic classification of OCD is based on a list of signs and symptoms, in which the ones that are observable and measurable (indirectly or from a third-person perspective^21^) are prioritized, and their interrelational dynamics are not considered. The criteriological manuals delineate OCD defectively, which results in diagnoses of dubious validity, overdiagnosis, the pathologization of behaviors, and the overdiagnosis of comorbidities. The diagnostic criteria are chosen for practical, instrumental purposes according to their statistical prevalence. Although they are intended to be a-theoretical, they are inevitably selected according to the interests and values of the groups responsible for developing the diagnostic manuals. The fifth edition of the Diagnostic and Statistical Manual of Mental Disorders (DSM-V) has a new category for OCD and related disorders (OCRDs include body dysmorphic disorder, trichotillomania, hoarding disorder, skin-picking disorder), and removing it from the group of anxiety disorders (American Psychiatric Association, 2013). This category transition is controversial because it puts too much weight on the impairment of inhibitory executive control. It is a superficial, reductionist understanding that fails to take account of the complexity drafted in our description of the obsessive–compulsive existential type.

The Yale-Brown Obsessive–Compulsive Scale-II (YBOCS) is the gold standard structured interview for measuring severity and response to treatment in OCD in research settings (Goodman et al., 1989; Storch et al., 2010). However, like all psychiatric scales, it cannot capture all the subtleties of experience, which makes it open to criticism for potential shortcomings, reductionism, and limitations (Mataix-Cols et al., 2004; Parnas et al., 2013; De Haan et al., 2013b). Moreover, using the deductive method, statistical correlations are made between indirect variables (for example, criteriological diagnosis and psychometric tests or magnetic resonance images), and reductionist causal relationships are inferred from such correlations. It is an approach often adopted unquestioningly as if it were an absolute (Aragona, 2011). However, it fails to consider the complexity and interdependence of factors contributing to OCD and may lead to a narrow understanding of the disorder.

While operational diagnostic systems, structured interviews, and deductive inferences may have limitations, they are widely used and have been subject to extensive research. It is important to point out that we do not propose the phenomenological-dialectical approach as a replacement for the operational diagnostic approach. Both are complementary. They provide different perspectives in the evaluation of the same psychopathological phenomena. Our comprehension of the obsessive–compulsive existential type from the phenomenological-dialectical perspective could give pointers to overcome some limitations of the biological-cognitivist explanation of OCD.

Crucially, the dialectical-phenomenological approach prioritizes the immediate experience, that is, the patient’s reports (i.e., first-person perspective knowledge) and, above all, the intuitive apprehension that emerges from the patient-psychopathologist encounter (i.e., second-person perspective knowledge). The observer evaluates the experience by making movements of attachment and detachment to it. Clinical symptoms are expressions of a specific structure and can only be comprehended concerning the whole. This perspective allows a more nuanced understanding of psychopathological alterations and a refinement of nosological entities, resulting in greater validity. Furthermore, the dialectic between generalization and individualization enables better therapeutic plans to be developed to meet the needs of individual patients.

One way to qualitatively refine the assessment and measurement of OCD could be to merge some specific domains of phenomenological psychopathology—like those described in phenomenological interviews (Sholokhova et al., 2022)—with the well-established YBOCS scale. Similarly, Nelson et al. (2021) argue that a “high-resolution clinical phenotype” could be traced by integrating clinical-staging phenotypes with the domains of selfhood, embodiment, and affectivity. For example, combining clinical (Fontenelle and Yücel, 2019) and phenomenological OCD models (as proposed in our study) could yield fruitful results. Also, more refined categories and fine-tuned severity staging could help develop a stronger correlation with new neurobiological and neuroimaging findings that aim to explore enactivism, intersubjectivity, and embodiment.

### 5.2. Pharmacological treatments

The notion of anthropological disproportions could extend phenomenological inquiries to the field of pharmacology (Tamelini and Messas, 2019). Selective serotonin receptor inhibitors (SSRIs) are the first option in the pharmacological approach to OCD and are generally used in high doses (Stein et al., 2019). They are also effective for treating depressive and anxiety disorders, raising questions about their standard action. Phenomenologically, SSRIs increase distance, disconnection, and disengagement between the self and the world (Teal, 2009). Acutely, SSRIs work to chemically protect the boundaries of the self, operating as a buffer. In some ways, they also modulate bodily resonance (Svenaeus, 2007). Reports of affective flattening (highs and lows) are frequent among SSRI takers (Teal, 2009). Our analysis of the obsessive–compulsive existential type through the lens of dialectical phenomenological psychopathology would suggest as a preliminary proposal that, like rituals, SSRI could act as a counterbalancing movement to impose distance, albeit from without, and more rapidly. The external management of distance by SSRIs reduces sensitivity and responsiveness (reactivity) to the world, diminishes body tension, increases perspectivity, and expands the sense of owning life (Teal, 2009). Also, if prescribed early, there is evidence that they can be more effective (Varigonda et al., 2016).

In severe or refractory cases of OCD, atypical antipsychotics (mainly risperidone and aripiprazole) are also indicated (Stein et al., 2019). As previously expounded, the essence of obsession can distort the individual structure of the personality, allowing its constitution to approach that of psychosis (disrupted minimal self). Thus, this class of medication would work like a plaster cast, bolstering the stability of the existential structure, like the use associated with treating schizophrenia (Tamelini and Messas, 2019).

### 5.3. Psychotherapeutic treatments

Regarding psychotherapies, cognitive-behavioral therapy (CBT), which includes exposure and response prevention (ERP), is considered the most evidence-based form of OCD treatment (Stein et al., 2019). It enables the patient to tolerate distress and discomfort without engaging in compensatory attitudes. CBT can help patients gain perspective, more self-confidence, and self-ownership, perceive others, and relieve bodily tension (e.g., associated with mindfulness). Although statistical epidemiological studies with CBT have found it effective, issues like drop-out and relapse need to be understood better, and significant changes in patients’ behavior need to be analyzed (Kyrios et al., 2015), which is where our study could help. The statement that cognition mediates no more than conditioned behaviors is a shallow premise that can be deconstructed according to our explanation of the obsessive–compulsive positional sense (see 4). Obsessive thoughts are not misinterpretations that should be confronted and overcome (Francesetti, 2017). Inaccurately, CBT assumes that patients intentionally assign misadjusted meanings and that repetitive training can effectively change them. However, as we have seen, their sense-making is determined by their aprioristic structural arrangements. In addition, ERP is restricted to secondary defensive rituals only.

From our analysis, the goal should not be to abolish rituals; instead, the treatment should focus on recalibrating proportions, such as extending the patient’s lived space to widen their horizon of possibilities (Fuchs, 2007b). Other psychotherapeutic practices, which focus too much on interpreting thematic contents due to intra-psychic conflicts and neglect the phenomenological structure and dynamics of the altered experiences, may have even more limited therapeutic effectiveness (Gabbard, 2001; King, 2017). Therefore, our preliminary suggestion would be that psychotherapy must clarify structural self-incongruities and ambiguities and help patients accept and modulate them. Embarrassment and shame must be minimized to enhance treatment adherence. These proposals seem to be effective as has been addressed by studies of Acceptance and Commitment Therapy (ACT) and Compassion Focused Therapy (CFT; Petrocchi et al., 2020; Soondrum et al., 2022).

Family therapy and psychoeducation, in which strategies to avoid accommodation are taught, can significantly impact the treatment of obsessive–compulsive patients (Demaria et al., 2021). There is an association between family accommodation and greater severity and poorer response to treatment (Wu et al., 2016). According to our study, we hypothesize that a relative who does not engage in perpetuating rituals will keep open the possibility of the patient’s re-synchronization via intersubjectivity. Thus, a relative who refuses to be restricted to roles related to rituals can contribute to treating obsessive–compulsive patients because it compels them to exercise other role identities and discourages the fragile patient from acting as a parasite on another’s strength (need of reassurance). A suitable patient-therapist bond can work similarly in re-synchronization regardless of their theoretical background. The therapist must find an optimal and non-harmful interpersonal lived distance to overcome the obsessive–compulsive patient’s self-enclosure and then allow the partial fusion of the horizons of their respective worlds (Fuchs, 2007b).

## 6. Conclusion

According to the biological-cognitive model, obsessive–compulsive disorder is a neuropsychiatric nosological entity caused by neurochemical and neuroanatomical alterations in specific brain circuits of emotion regulation, inhibitory executive control, habituation, and reward system. Its diagnosis can be made simply by identifying a cluster of symptoms and behaviors. However, this prevailing paradigm has been questioned and critiqued for its lack of ontological concern, mechanistic and reductionist theoretical framework, methodological limitations (inferences from indirect statistical correlations ensuring only internal consistency), weak validity, and few contributions to better mental healthcare. In this context of the paradigm crisis, there has been a resurgence of interest in the phenomenological method, to which our study contributes. Specifically, we propose a return to the raw obsessive–compulsive experience and identifying its fundamental constituents, arranged according to the dynamic dialectic of anthropological proportions. The obsessive–compulsive existential type is a global transformation of existence that can be grasped in the concrete clinical encounter between psychopathologists and patients. Its clinical presentations result from the complex dynamic interplay of the supremacy of the *anti-eidos* (primary alteration) and the resulting compensatory mechanisms, which attempt to maintain structural balance in the existential structure (summarized in Figure 5). Obsessive–compulsive patients are subject to an annihilating existential force, yet they are simultaneously the source of this process of degradation, expressed in the deregulated compensatory process.

The study of altered experience statically searching for the grasp of a fixed psychopathological essential core has shown to be limited, especially in the case of OCD. The dialectical study was merged with the phenomenological approach to address the dynamic aspect of psychopathological presentations. The study of dialectics enables the dynamics of anthropological disproportions to be understood. In the obsessive–compulsive condition, as described in the present study, the simplest of tendencies toward the predominance of one polarity over the other already presents clinically heterogeneous symptoms. Moreover, any such movement tends to be counterbalanced in another to regain equilibrium. The proposed kinetic analysis of the dialectics of anthropological disproportions in the obsessive–compulsive existential type can provide a more encompassing, nuanced, and experience-faithful comprehension of the variability and heterogeneity of this condition, helping to improve staging, validity, and differential diagnosis. In this way, this approach can also be used to suggest some refinements to pharmacological, psychotherapeutic, and biological therapeutic strategies. However, it is essential to note that our study does not aim to provide an exhaustive, finished comprehension of the phenomenon or any dogmatic solutions for the clinical management of the obsessive–compulsive existential type. Rather, loyal to the phenomenological tradition, our goal is to contribute to the ongoing dialogue and deepen the epistemic iteration of this complex and intriguing topic without rejecting the buildings of knowledge from other perspectives.

## Data availability statement

The original contributions presented in the study are included in the article/supplementary material, further inquiries can be directed to the corresponding author.

## Author contributions

LF contributed to the design, writing, and discussion of the manuscript. MT contributed to the design and discussion of the manuscript. GM contributed to the discussion of the manuscript. All authors approved the submitted version.

## Funding

LF was supported by Coordenação de Aperfeiçoamento de Pessoal de Nível Superior—Brazil CAPES/PROSUP.

## Conflict of interest

The authors declare that the research was conducted in the absence of any commercial or financial relationships that could be construed as a potential conflict of interest.

## Publisher’s note

All claims expressed in this article are solely those of the authors and do not necessarily represent those of their affiliated organizations, or those of the publisher, the editors and the reviewers. Any product that may be evaluated in this article, or claim that may be made by its manufacturer, is not guaranteed or endorsed by the publisher.

## Footnotes

^1^The term “life-world” is also used to refer to this coherent gestalt (Stanghellini and Mancini, 2017).

^2^Phenomenological compensations result from the ways consciousness restructures itself to guard against primary impositions (Minkowski, 1933/1995; Sass, 2010).

^3^This division is merely didactic since all categories are interrelated.

^4^We are aware that the way we use some phenomenological psychopathological concepts does not fully align with the sense in which they are used by some philosophers. For instance, from a rigorous philosophical perspective, switching from an existentialist to a Husserlian referential may seem inconsistent in dealing with temporality. However, we build on Basso’s understanding that psychopathologists traditionally based their concepts on philosophical ones as long as they were useful and coherent for understanding clinical situations (Basso, 2009), i.e., as they follow the rationale of the psychopathological independent scientific field. This perspective is also supported by Messas (2021). Hence, as we will see, the almost geometric Husserlian conception of time flow can depict the temporal slowdown and rearrangements in the obsessive–compulsive patient in a very fruitful manner.

^5^This temporal variability can also substantiate the heterogeneity of clinical presentations.

^6^Intentionality has a temporal structure, i.e., we direct ourselves toward objects and goals meaningfully, shaped by past experiences and anticipations. As a result, changes in how experiences are synthesized over time can affect intentionality (Fuchs, 2007a).

^7^For example, the act of drinking water (overall action) is constituted by walking to a water fountain, picking up a cup, turning on the faucet, and moving one’s hand to one’s mouth, swallowing, these are all acts of volition and directed. But these partial steps are not fully clear to our consciousness. Some steps have a clearer purposeful goal (e.g., walking to a water fountain), while others seem to be more linked to habitual body memory (e.g., swallowing). In normal context what we are aware of is simply the overall act of drinking water and feeling satisfied (fulfillment of the expectation).

^8^The analogy to stammering is a useful metaphor for representing this aspect (Gibbs, 2010). The repetition of syllables may not change the overall directionality of speech, but it does impair its fluidity. If the problem becomes more severe, the speech may splinter and fall apart.

^9^Within this debate, the concept of “schizo-obsessive disorder” has emerged.

^10^Straus defines “*pathic*” as “a characteristic feature of primordial experience”; “the immediately present, sensually vivid, still pre-conceptual (pre-cognitive) communication we have with appearances” (Straus, 1935; Ratnapalan and Reggio, 2012, p. 296). We drew a parallel between Straus’s “pathic” aspect and Tellenbach’s concept of affective atmosphere (Tellenbach, 1985), which describes the emotional and affective qualities that permeate a person’s experience of the world.

^11^The historical discussion about which psychic functions are primarily altered in obsessions has its roots here. In general, the German school (Griesinger and Westphal) prioritized the cognitive aspect and the French school (Morel and Janet) prioritized the emotional aspect (Berrios, 2008). The view of one school is not predominant over the other. What happens is that each one has elected particular frames to reveal the same structural configuration. What is also observed in clinical practice is that in some cases, the affective aspect is more exuberant, while in others there is a predominance of altered intellectual function. This can explain the variability of obsessive–compulsive clinical presentations between neurosis/psychosis (Lang, 1985) and personality development/process (Jaspers, 1913/1997).

^12^This fragility was also historically revealed in psychasthenia (Janet, 1903).

^13^Intolerance of uncertainty has been identified as one of the primary cognitive factors that contribute to the development and maintenance of the OCD (Kalanthroff et al., 2017; Shephard et al., 2021). This aspect will be developed in the section 4.

^14^Embarrassment and shame are complex emotions that not only display our pre-reflective and reflective self-awareness but also reveals our relationality (Zahavi, 2015).

^15^Interestingly, some studies suggest the management of OCD sensory phenomena with ondansetron (a 5-HT3 receptor antagonist, FDA-approved for severe nausea and vomiting; Shephard et al., 2021).

^16^This is not unlike the experience of sensitive patients described by Kretschmer in *The Sensitive Delusion of Reference* or the entity named Olfactory Reference Syndrome.

^17^We are not referring to the notion of defense as understood in psychoanalysis. In psychoanalysis, defenses are unconscious psychological actions of the ego that seek to relieve anguish or protect it from frustrations and conflicts. Structural rearrangements have a quality of non-deliberated effort, given that occur in the immanent conditions of possibility of all lived experiences.

^18^Based on this, we can understand how the heterogeneity of clinical presentations of obsessive–compulsive clinical presentations stems from this potential for variability in structural arrangements and dynamics, both within the same patient longitudinally and across multiple patients.

^19^Sisyphus is a character in Greek mythology condemned eternally to repeatedly roll a huge rock up a hill only to have it roll down again as it nears the top.

^20^For more details on obsessive–compulsive personality disorder (OCPD)—anankastic personality—we recommend study on the anthropopathology of obsessive disproportion of Messas (2021).

^21^The psychopathologist is an observer detached from the experience.
